# Impact of age and socioeconomic status on treatment and survival from aggressive lymphoma: a UK population-based study of diffuse large B-cell lymphoma

**DOI:** 10.1016/j.canep.2015.08.015

**Published:** 2015-12

**Authors:** Alexandra Smith, Simon Crouch, Debra Howell, Cathy Burton, Russell Patmore, Eve Roman

**Affiliations:** a Epidemiology and Cancer Statistics Group, Department of Health Sciences, University of York, YO10 5DD, UK; b St James’s Institute of Oncology, Leeds Teaching Hospitals NHS Trust, LS9 7TF, UK; c Queens Centre for Oncology, Castle Hill Hospital, HU16 5JQ, UK

**Keywords:** Non-Hodgkin lymphoma, Diffuse large B-cell lymphoma, Inequality, Chemotherapy, Age, Socio economic status

## Abstract

**Aim:**

To examine the influence of patient’s age and socio-economic status on treatment and outcome in diffuse large B-cell lymphoma (DLBCL); an aggressive curable cancer, with an incidence rate that increases markedly with age but varies little with socio-economic status.

**Methods:**

Set within a representative UK population of around 4 million, data are from an established patient cohort. This report includes all patients (≥18years) newly diagnosed with DLBCL 2004–2012, with follow-up to February 2015.

**Results:**

Of the 2137 patients (median age 70.2 years) diagnosed with denovo DLBCL, 1709 (80%) were treated curatively/intensively and 1161(54.3%) died during follow-up. Five-year overall and relative survival (RS) estimates were 46.2% (95% CI 44.0–48.4%) and 54.6% (52.1%-57.0%) respectively for all patients, and 58.5% (56.1–60.9%) and 67.0% (64.3–69.6%) for intensively treated patients. 96.3% of patients <55 years (366/380) and 96.4% of those with the best performance status (543/563) were treated curatively: 5-year RSs being 77.9% (73.1–82%) and 87.1% (82.5–90.6%) respectively. At the other end of the age/fitness spectrum, 33.3% of those ≥85 years (66/198) and 41.1% with the worst performance (94/225) were treated curatively: the corresponding 5-year RSs being 50.5% (27.1–69.0%) and 22.9% (14.0–33.2%). The proportion of patients whose cancer was fully staged fell with increasing age and worsening performance status. No socio-economic variations with treatment, stage at presentation or outcome were detected.

**Conclusions:**

Performance status is more discriminatory of survival than chronological age, with fitter patients benefiting from treatment across all ages. Socio-economic factors are not predictive of outcome in patients with DLBCL in the UK.

## Highlights

• Age and performance status were predicative of treatment and survival.
• Sixty percent of patients ≥75 yrs were treated with curative intent.
• Performance status was more discriminatory of treatment and survival than age.
• Socio-economic factors were not predictive of survival.
• Clinical characteristics aide interpretation of socio-demographic treatment/outcome trends.

## 1 Introduction

More than half of all cancers are diagnosed in those aged 70 years or over in developed regions of the world; and this proportion is growing as life expectancy increases and populations age [1], [2], [3]. That older cancer patients may be offered less intensive treatments than their younger counterparts is well known; and although this may be an informed and appropriate decision, there is concern that in some cases there may be over reliance on chronological age as a proxy for other factors which may, or may not, be present [4], [5], [6]. Moreover, it has been suggested that under-treatment of older people could, at least in part, explain the disparities in cancer survival observed both within and between countries with seemingly similar health care systems [7], [8], [9]. In this regard, UK cancer services have been at the centre of many of these discussions; with particular concerns being raised about equity in the provision of chemotherapy for potentially curable cancers [6], [7], [9], [10].

In addition to age, there is continued debate about the role that socioeconomic factors play in determining cancer treatments and outcomes [11], [12], [13], [14], [15], [16]. The underpinning reasons for such health inequalities are diverse and complex; both in countries like the UK that have universal health care coverage, and in countries like the USA that do not [17], [18]. In both situations, differentials in general health and stage at cancer presentation are likely to contribute to any trends observed; with adequacy of personal insurance coverage playing an additional role in countries where individuals have to pay for their care at the point of delivery [15], [19]. However, as with questions about age biases, the socioeconomic determinants of cancer treatment and survival in the UK continues to be a topic of public concern and scientific interest; with recent evidence suggesting that the persistent differentials seen for many common cancers may, in fact, be widening [20].

With standardized chemotherapy, and an incidence rate that does not vary systematically with markers of socio-economic status but increases exponentially after the age of 55 years [21], [22], diffuse large B-cell lymphoma (DLBCL) is an exemplar cancer within which to examine treatment and survival variations. DLBCL is the commonest of the haematological malignancies (leukaemias, lymphomas and myelomas), accounting for around 48% of all non-Hodgkin lymphomas [23]. CHOP (cyclophosphamide, doxorubicin, vincristine, and prednisone) has been the staple chemotherapy for DLBCL for the last 35 years; the addition of the monoclonal anti-CD20 antibody Rituximab (R-CHOP) in 2003 increasing the overall 5-year survival to around 60% . However, whilst R-CHOP can be effective at any age, increasing levels of frailty and comorbidity, as [24], [25] well as decreasing ability to tolerate the side-effects of intensive chemotherapy, mean that increasing age remains associated with poorer outcome [24], [25].

## 2 Methods

Data are from the UK’s population-based Haematological Malignancy Research Network (www.hmrn.org) which, with a catchment population of nearly 4 million people, has a socio-demographic composition that broadly mirrors that of the UK as a whole. Initiated in 2004, full details of its structure, data collection methods and ethical approvals have been previously described [26]. Briefly, within HMRN patient care is provided by 14 hospitals organized into five multi-disciplinary teams (MDTs); and clinical practice adheres to national guidelines. As a matter of policy, all diagnoses across the HMRN region are made and coded by clinical specialists at a single integrated haematopathology laboratory—the Haematological Malignancy Diagnostic Service (www.hmds.info); cited in the UK’s Department of Health’s Cancer Reform Strategy as “the model for delivery of complex diagnostic services”[27]. HMRN operates with Section 251 support under the NHS Act 2006, and all patients have prognostic, full-treatment, response and outcome data collected to clinical trial standards. All newly diagnosed patients are ‘flagged’ and followed-up for death and subsequent cancer registrations at the national Medical Research Information Service (MRIS) and routinely linked to nationwide information on Hospital Episode Statistics. Area-based population counts and measures of deprivation are sourced from the Office for National Statistics [21], [28].

The present report includes all patients (≥18 years) newly diagnosed with denovo DLBCL (*n* = 2137) between 1st September 2004 and 31st August 2012; all of whom were followed-up until the 6th February 2015, with primary source information on presentation, treatment and management including chemotherapy regimen being obtained directly from medical records. In accordance with national guidance and other epidemiological studies [29], [14], [16], the standard measure - income domain of the national index of deprivation (IMD) [30]—was used as a marker of socio-economic status; quintile one containing the most affluent fifth of England’s lower super output areas and quintile five the least. Information on cancer stage and patients performance status were also used in the analysis: non-Hodgkin lymphomas being staged using the modified Ann Arbor system [31], and performance status graded using the Eastern Oncology Cooperative Group’s (ECOG) scale [32]. These scores, along with the indicators used to assess the presence of disease associated symptoms (B symptoms) are defined in Box 1.

All analyses were conducted using standard analytical methods in the statistical packages Stata 13 [33] or R [34]; odds ratios were estimated using logistic regression and time to event analyses by Cox proportional hazards regression models. The Stata program strel (v1.2.7) was used to estimate relative survival, which is based on the maximum likelihood method for individual records developed by Estève et al [35]; with age and sex-specific background mortality rates being obtained from national life tables [36]. Due to the large number of lymphoma-related deaths in the first year following diagnosis, survival probabilities were initially estimated for monthly intervals and progressively increased up to yearly intervals until 5-years after diagnosis. In order to assess the ability of age and performance status to predict treatment, the receipt of curative chemotherapy was treated as a binary outcome in logistic regression with age, performance status, and stage included as explanatory variables. The ability of each model to predict the receipt of chemotherapy was assessed by calculating the area under the curve (AUC) of the corresponding receiver operator curve (ROC).

## 3 Results

The demographic and clinical characteristics of the 2137 patients (≥18 years) diagnosed with DLBCL over the eight year period September 2004-August 2012 are stratified according to whether or not they received intensive first-line chemotherapy with curative intent in Table 1. In total, 1709 (80.0%) patients received such chemotherapy and 428 (20.0%) did not, either because they died before such treatment could be initiated or the decision was taken to manage their disease with a palliative approach, with radiotherapy only or with single agent chemotherapies such as vincristine. Of the patients who received intensive treatment, 85% were treated with R-CHOP, the remainder were mainly treated with R-CVP and R-CODOX-M/R-IVAC. The proportion of patients receiving intensive chemotherapy with curative intent varied markedly with three interconnected characteristics; falling with increasing age (*P *< 0.0001), worsening performance status (*P *< 0.0001), and increasing cancer stage (*P *< 0.0001). By comparison, the association with the presence of B symptoms was weak (adjusted Odds Ratio = 1.34, 95% CI 1.03–1.75); and no associations between intensive chemotherapy administration by sex or area-based deprivation were detected.

**Fig. 1:**
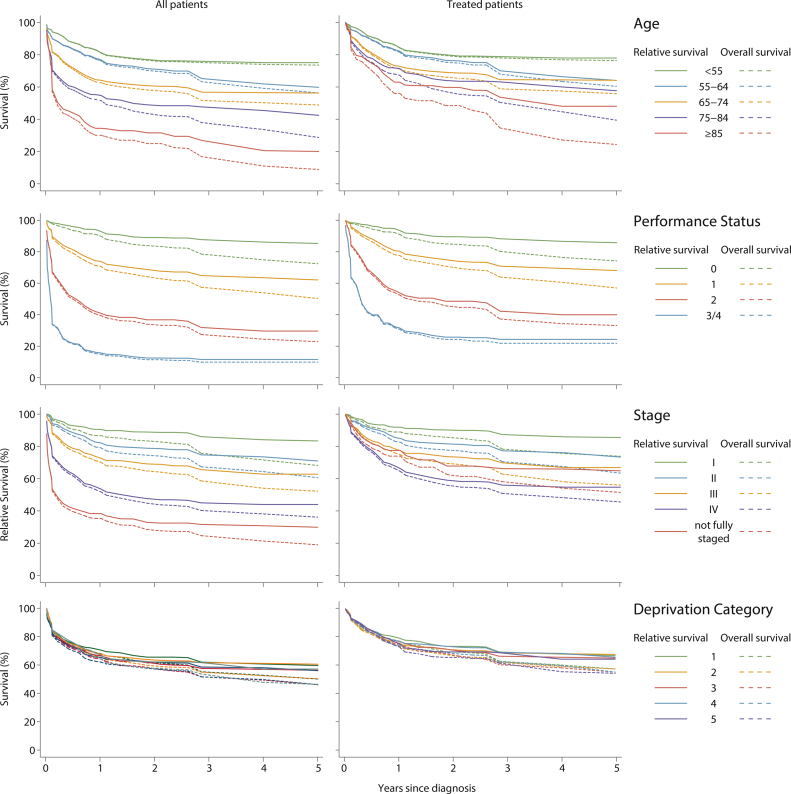
Overall and relative survival curves by age, performance status, stage and deprivation for all patients and chemotherapy treated patients: HMRN patients (≥ 18 years) diagnosed with DLBCL 2004–12 and followed until February 2015.

Just over half (1161/2137) of the patients died during the follow-up period (Table 2): the 5-year overall and relative survivals being 46.2% (95% CI 44.0–48.4%) and 54.6% (95% CI 52.1–57.0%) respectively (Table 3). Patients treated with intensive chemotherapy had better survival than the totality, the 5-year overall and relative estimates increasing to 58.5% (95% CI 56.1–60.9%) and 67.0% (95% CI 64.3–69.6%) respectively. Age, performance status, and stage were strongly predicative of outcome; the discrimination being clearest for performance status, both among all patients and among patients treated with curative intent (Table 2, Table 3, Fig. 1). By contrast, no associations with deprivation were observed. Our findings are discussed in more detail in the sections below.

### 3.1 Age at diagnosis & performance status

The proportion of patients treated with curative intent fell gradually from 96.3% (366/388) in under 54 year olds to 86.8% (537/619) in 65–74 year olds, before falling more steeply to reach 69.9% (386/552) in 75 to 84 year olds and 33.3% (66/198) in those aged 85 years or more (Table 1). The pattern with performance status followed a more linear trend, falling incrementally from 96.4% (543/563) in those with a performance of 0 through to 32.2% (19/59) in those with a performance status of 4.

**Fig. 2:**
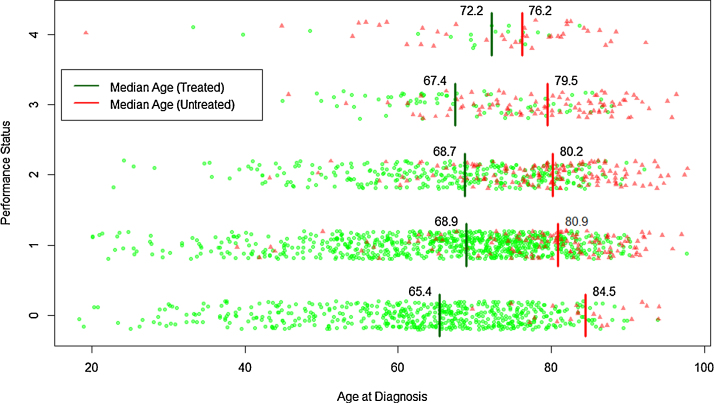
Jitter plot showing the patients distributed by performance status and age according to whether they received chemotherapy (green dots, with median ages marked with a green bar) or not (red triangles, with median ages marked with a red bar): HMRN patients (≥18 years) diagnosed with DLBCL 2004–12. (For interpretation of the references to color in this figure legend, the reader is referred to the web version of this article.)

The impact of age and performance status on the administration of chemotherapy with curative intent is shown more clearly in the jitter plots in Fig. 2: patients receiving chemotherapy are marked as green dots and those who did not as red triangles. Among patients whose performance status was zero, age was highly predictive of non-receipt of chemotherapy (AUC = 94% for a simple logistic model); and, with a median diagnostic age of 84.5 years, the 20 patients who did not receive chemotherapy were, on average, older than any other group. Our core abstraction forms indicate that ten of these patients had a recorded entry in their medical notes stating their preference to decline intensive treatment.

Age was less predictive of non-receipt of chemotherapy among patients whose performance status was greater than zero; the AUCs for simple logistic regression being 78%, 78%, 75% and 62% respectively for categories one through to four. The varying effect of age by performance status was confirmed in logistic regression with an interaction between age and performance status (*P* = 2.5 × 10^−6^ in LR test versus a main effects only model). As can be seen from Fig. 2, the median age at diagnosis fell as performance status worsened; from 84.5 years among those in category zero, through to 76.2 years among those in category four. By contrast, among those who received chemotherapy, median age increased with deteriorating performance status from 65.4 years in those who were category zero through to 72.2 years in those who were category four. The reasons for non-receipt of chemotherapy among younger patients with performance status one to four were very diverse; and included factors such as the presence of sepsis, serious co-morbidities, patient refusal, and death before treatment could be initiated.

**Fig. 3:**
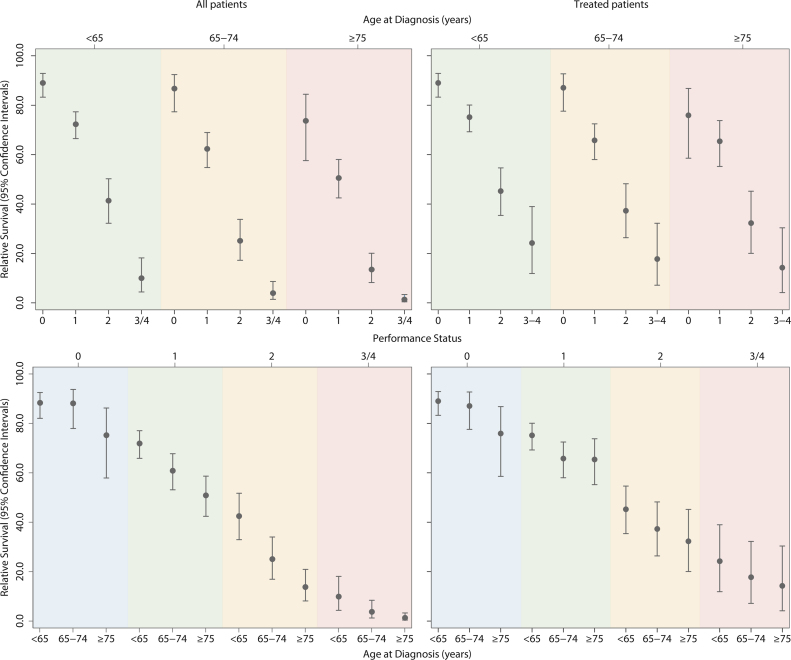
5-year relative survival estimates and 95% confidence intervals stratified by age and performance status for all patients and chemotherapy treated patients: HMRN patients (≥18 years) diagnosed with DLBCL 2004–12 and followed until February 2015.

Five-year overall and relative survival estimates for all patients and those treated with curative chemotherapy are distributed by patient characteristics in Table 3. The 5-year RS of the 96.3% (366/380) of patients <55 years who were treated curatively was 77.9% (95% CI 73.1–82%), and that of the 96.4% (543/563) with a performance status of zero who were also treated curatively was 87.1% (95% CI 82.5–90.6%). At the other end of the age and fitness scales, 33.3% (66/198) of those ≥85 years and 41.8% (94/225) of those with a performance status of 3/4 were treated curatively: the corresponding 5-year RSs being 50.5% (95% CI 27.1–69.0%) and 22.9% (14.0–32.2%) respectively. That the relationship between performance status and survival is broadly similar within all age strata is illustrated more clearly by the 5-year relative survival estimates shown in the top panel of Fig. 3. The importance of performance status is further evidenced in the bottom panel of Fig. 3, where the 5-year relative survival estimates are stratified by age within individual categories.

#### 3.1.1 Age at diagnosis, stage and deprivation

Two-hundred and fifty patients (11.7%) did not have all of the investigations required to fully assign stage (Table 1). Staging of DLBCL requires a bone marrow biopsy as well as a CT and/or PET scan; and the proportion who did not have all of these investigations increased markedly after the age of 75 years, accounting for 42% of the total in those aged 85 years or more (Supplementary Fig. 1). Furthermore, patient’s performance status and cancer stage are strongly correlated; with those whose cancer was not fully staged also tending to have poor performance status. By contrast, there is no strong evidence of a relationship between stage at presentation and deprivation. In addition, no association between performance status and deprivation was observed (data not shown).

Supplementary material related to this article found, in the online version, at http://dx.doi.org/10.1016/j.canep.2015.08.015.

**Figure:**
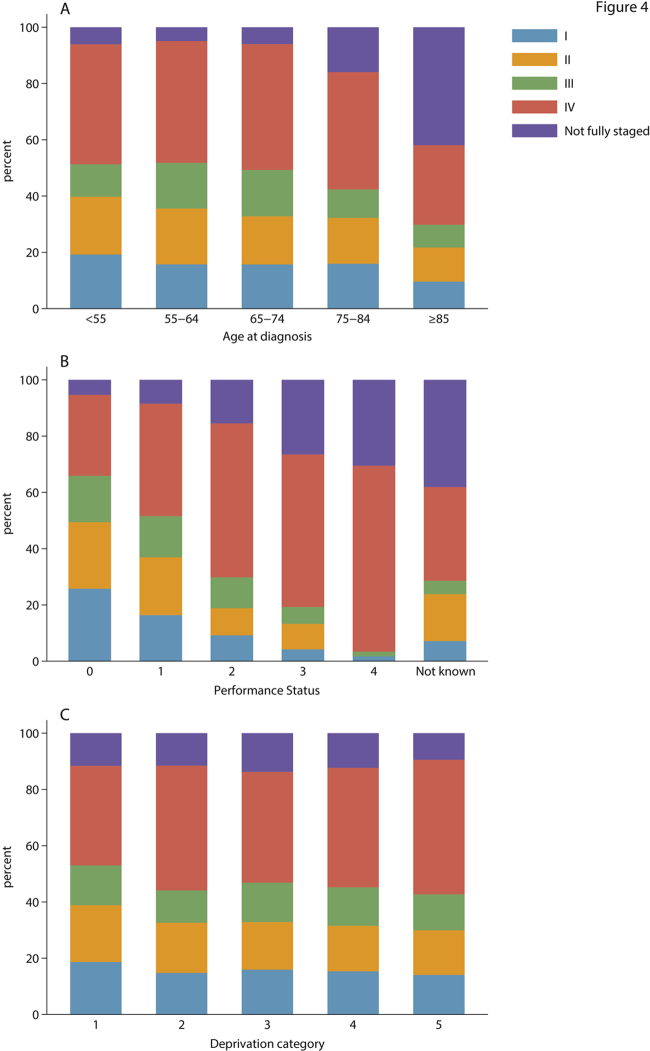
Supplementary Fig. S1 Cancer stage by A) age at diagnosis (years) B) performance status C) deprivation: HMRN patients (≥ 18 years) diagnosed with DLBCL 2004-12.

## 4 Discussion

With a median diagnostic age of 70 years, our UK population based study of 2137 patients newly diagnosed with the aggressive but curable cancer, diffuse large B-cell lymphoma (DLBCL), found that general fitness, as measured by performance status, was more discriminatory of survival than chronological age; with comparatively fit patients treated with curative intent benefitting across all age groups. Furthermore, in contrast to cancers that have strong environmental/life-style risk factors and/or screening programmes—such as breast, lung and colorectal cancers—area-based deprivation was not found to be predictive either of stage at presentation or of survival. Somewhat paradoxically, the strongest association between chronological age and treatment with intensive chemotherapy was seen among the 563 patients with the best performance status; where the 96.4% of patients treated with curative intent were, on average, younger than any other group (median age 65.4 years) and the 20 patients who did not receive such treatment were, on average, the oldest (median age 83.5 years). However, at least 10 of the 20 patients in this latter group declined intensive treatment; and in this regard it is important to note that shared-decision making is a key clearly defined component of UK healthcare policy, with emphasis placed on the patient as the final arbiter of the management approach that best suits their preferences, even if this is to decline treatment [37], [38].

Using the same commonly applied index of multiple deprivation as a marker of socio-economic status as used here, we have previously demonstrated significant survival inequalities within our catchment population for chronic myeloid leukaemia (CML) [39]; a once rapidly fatal cancer transformed in the early 2000s by orally administered tyrosine kinase inhibitors into a long-term condition with a steadily rising prevalence. Unlike CML, which is controlled by lifelong daily therapy, patients with DLBCL who survive intensive chemotherapy are considered cured; with those who are not treated curatively and those who do not respond to chemotherapy tending to die within a few months of diagnosis. Hence, the drivers for the socio-economic variations seen within our population for CML are likely to be very different from those that could potentially impact on DLBCL.

Whilst no evidence of socio-economic inequalities in stage at presentation, treatment or survival for DLBCL was found in our UK population, differences have been reported from elsewhere; most notably from the USA where pronounced survival disparities associated with insurance status have been described for many cancers, including DLBCL [19], [40], [41]. Contemporary socio-economic data on DLBCL from Europe, where personal health insurance does not exert the same influence as in the USA, are sparse. However, the most recent report from Denmark, which included almost 90% of all lymphoma diagnoses 2000 to 2008, noted elevated mortality among DLBCL patients of lower socio-economic status; the authors concluding that delayed presentation may have had a role to play [42]. That we failed to detect such differences in our more recent data (diagnoses 2004 to 2012) could, at least in part, be due to the survival improvements generated by the introduction of Rituximab in 2003. Rituximab, which was trialled in patients aged 60–80 years because of its low toxicity [43], has impacted on DLBCL survival across all ages and cancer stages; the outcome for patients with delayed presentation and more advanced disease being much better now than it was in the past [24], [25]. Indeed, in our data whilst patients’ age and general fitness, as measured by their performance status, were strongly discriminatory of both intensive chemotherapy and survival, a positive impact on outcome was clearly evident among older patients who received curative treatment: the 5-year relative survival estimates of those surviving the first months of treatment paralleling those of the general population. Undoubtedly, the emergence of novel targeted agents like Rituximab has drawn attention to the fact that the age dichotomizations used in traditional prognostic scores are no longer as informative as perhaps they once were [24], [25]. In this regard, as well as the requirement for less toxic and more effective treatments, there is a clear need for better tools to predict an individual’s tumour response and their ability to tolerate therapy.

Examining and interpreting socio-demographic differentials is always challenging, particularly in fast-moving areas of oncology where treatment protocols are subject to rapid change, and ‘gold standard’ randomized controlled chemotherapy trials are often restricted to specific patient groups; traditionally younger patients with fewer co-morbidities. The ability to conduct comprehensive population-based analyses of the type presented here is, however, a fundamental attribute of the UK’s NHS. Predicated on these structures, our population-based patient cohort was initiated to produce ‘real-world’ generalizable data to inform contemporary clinical practice and research; major strengths including its large well-defined catchment area, completeness of ascertainment and world-class diagnostics. Importantly, the socio-demographic structure of our catchment population, which at around 4 million accounts for around 6% of the UK’s estimated total, is broadly representative of the national population in terms of age, sex, and deprivation; and clinical practice adheres to national guidelines [21], [26]. Crucially in this respect, because all diagnoses within HMRN are made and coded by clinical experts, our data do not suffer from the problems commonly encountered by non-specialist registries, where lymphomas are often registered using not otherwise specified (NOS) morphology codes, such as lymphoma NOS (9590) or non-Hodgkin lymphoma NOS (9591) [44]. In practice this means that cancer registry sub-type frequencies can be implausibly low; a recent analysis of routine cancer registrations in the UK reporting, for example, that DLBCL accounted for only 26% of all non-Hodgkin lymphomass—far less than the 48% recorded in our specialist registry [23]. Furthermore, our use of clinical data relating to performance status, cancer stage and presence of B-symptoms serves to highlight the importance of incorporating such information into studies examining the impact of socio-demographic factors on treatment patterns and survival.

In summary, although patient’s age and performance status (fitness) were predictive of both intensive chemotherapy and survival; performance status was far more discriminatory of outcome than age, with fitter patients benefiting from treatment across all age groups. Furthermore, in the UK setting of universal health-care coverage, we found no evidence that socio-economic factors were predictive of DLBCL stage at presentation, treatment or survival. In this regard, data from the Benchmarking Partnership 1995-2007, confirmed that UK survival for breast, colorectal, lung and ovarian cancer lagged behind that reported for Australia, Canada, Norway and Sweden [9]. However, with 80% of cancer patients in our study being treated with curative intent, our 5-year relative survival estimates for DLBCL are broadly comparable to those of other European countries [45], [46]. Whilst this could be due to the fact that UK cancer services have improved in recent years, it is also possible that the national survival differences seen for many cancers may not extrapolate uniformly to all. Accordingly, future comparative analyses of survival may benefit from the inclusion of potentially curable cancers, such as DLBCL, which do not have strong environmental determinants to their aetiology.

## Conflict of interest

None of the authors have any conflicts of interest.

## Authorship contribution

ER, AS, and RP were responsible for the conception and design of the study. DH and AS supervised data collection, with AS linking and managing all of the data. AS and SC carried out all of the analyses, with SC taking responsibility for the statistical methods employed. CB and RP provided clinical advice regarding the analysis and interpretation of the findings. ER and AS are the study guarantors and take responsibility for the integrity of the data. All authors contributed to the final draft of the paper; and have had full access to all of the data in the study.

## Funding

Leukaemia Lymphoma Research.

## Figures and Tables

**Table 1:** Numbers of patient and lymphoma characteristics distributed by first-line chemotherapy: HMRN patients (≥18 years) diagnosed with DLBCL 2004–12

		All patients	First line chemotherapy with curative intent		Odds ratio (95% Confidence Intervals)	Adjusted^a^ odds ratio (95% Confidence Intervals)
			Yes (%)	No (%)		
	Total	2137	1709 (80.0)	428 (20.0)		

Age at diagnosis (years)	18–54	380	366 (96.3)	14 (3.7)	3.99 (2.23–7.15)	3.82 (2.04–7.14)
55–64	388	354 (91.2)	34 (8.8)	1.59 (1.04–2.42)	1.38 (0.86–2.20)
65–74	619	537 (86.8)	82 (13.2)	1	1
75–84	552	386 (69.9)	166 (30.1)	0.35 (0.26–0.47)	0.32 (0.23–0.45)
≥85	198	66 (33.3)	132 (66.7)	0.08 (0.05–0.11)	0.07 (0.05–0.11)
Median (range)	70.2 (18.3–97.8)	67.4 (18.3–97.7)	80.4 (19.2–97.8)		
Trend χ^2^ (*P*-value)				379.8 (<0.0001)	281.1 (<0.0001)

Sex	Males	1117	919 (82.3)	198 (17.7)	1	1
Females	1020	790 (77.5)	230 (22.5)	0.74 (0.60–0.92)	1.05 (0.86–1.37)
Trend *χ*^2^ (*P*-value)				7.74 (*P *= 0.005)	0.13 (*P* = 0.72)

Patient performance status^c^	0	563	543 (96.4)	20 (3.6)	4.35 (2.68–7.08)	3.56 (2.10–6.02)
1	861	742 (86.2)	119 (13.8)	1	1
2	446	308 (69.1)	138 (30.9)	0.36 (0.27–0.47)	0.43 (0.31–0.60)
3	166	75 (45.2)	91 (54.8)	0.13 (0.09–0.19)	0.18 (0.12–0.28)
4	59	19 (32.2)	40 (67.8)	0.08 (0.04–0.14)	0.09 (0.04–0.17)
Not known	42	22 (52.4)	20 (47.6)	0.18 (0.09–0.33)	0.24 (0.11–0.53)
Trend χ^2^*P*-value				343.0 (<0.0001)^b^	178.8 (<0.0001)^b^

Lymphoma stage^c^	I	338	306 (90.5)	32 (9.5)	2.72 (1.83–4.05)	2.23 (1.44–3.44)
II	375	357 (95.2)	18 (4.8)	5.65 (3.43–9.31)	5.94 (3.42–10.30)
III	281	262 (93.2)	19 (6.8)	3.93 (2.40–6.42)	3.25 (1.92–5.49)
IV	893	695 (77.8)	198 (22.2)	1	1
Not fully staged	250	89 (35.6)	161 (64.4)	0.15 (0.11–0.21)	0.29 (0.20–0.42)
Trend *χ*^2^*P*-value				378.6 (<0.0001)^b^	171.3 (<0.0001)^b^

B-symptoms^c^	No	1182	939 (55.0)	243 (56.6)	1	1
Yes	955	770 (45.1)	185 (43.2)	1.07 (0.86–1.32)	1.34 (1.03–1.75)
*χ*^2^*P*-value				0.40 (0.53)	4.9 (0.03)

Deprivation (quintile)	1 (affluent)	466	383 (82.2)	83 (17.8)	1.06 (0.76–1.47)	1.09 (0.73–1.63)
2	494	402 (81.4)	92 (18.6)	1	1
3	414	325 (78.5)	89 (21.5)	0.82 (0.60–1.14)	0.82 (0.55–1.22)
4	365	283 (77.5)	82 (22.5)	0.79 (0.57–1.10)	0.73 (0.49–1.10)
5 (deprived)	391	312 (79.8)	79 (20.2)	0.90 (0.65–1.26)	0.71 (0.47–1.07)
Not known	7	4 (57.1)	3 (42.9)	–	–
Trend *χ*^2^*P*-value				4.2 (0.38)	6.3 (0.18)

a Adjusted for all other factors in the table.

b Excludes: not known/not fully staged.

c See definitions in Box 1.

**Table 2:** Numbers of deaths, person years and Hazard ratios (HR) distributed by patient, lymphoma and chemotherapy characteristics: HMRN patients (≥ 18 years) diagnosed with DLBCL 2004–12 and followed until February 2015.

		All patients					First line chemotherapy with curative intent				
		Total	Person years	Alive (%)	Dead (%)	Adjusted^a^ HR (95% Confidence Intervals)	Total	Person years	Alive (%)	Dead (%)	Adjusted^a^ HR (95% Confidence Intervals)
	Total	2137	7215	976 (45.7)	1161 (54.3)		1709	6915	957 (56.0)	752 (44.0)	
Age at diagnosis (years)	18–54	380	1792	278 (73.2)	102 (26.8)	0.43 (0.35–0.54)	366	1792	278 (76.0)	88 (24.0)	0.46 (0.36–0.58)
55–64	388	1562	223 (57.5)	165 (42.5)	0.74 (0.61–0.89)	354	1524	219 (61.9)	135 (38.1)	0.78 (0.63–0.96)
65–74	619	2181	291 (47.0)	328 (53.0)	1	537	2146	289 (53.8)	248 (46.2)	1
75–84	552	1405	165 (29.9)	387 (70.1)	1.66 (1.43–1.93)	386	1286	154 (39.9)	232 (60.1)	1.53 (1.28–1.83)
≥85	198	274	19 (9.6)	179 (90.4)	2.10 (1.74–2.54)	66	168	17 (25.8)	49 (74.2)	2.01 (1.48–2.73)

Sex	Male	1117	3766	509 (46.5)	608 (54.4)	1	919	3641	500 (54.4)	419 (45.6)	1
Female	1020	3453	467 (45.8)	553 (54.2)	0.89 (0.79–1.01)	790	3274	457 (57.9)	333 (42.1)	0.82 (0.71–0.94)

Patient performance status^b^	0	563	2731	410 (72.8)	153 (27.2)	0.54 (0.45–0.65)	543	2678	405 (74.6)	138 (25.4)	0.60 (0.49–0.74)
1	861	3350	429 (49.8)	432 (50.2)	1	742	3200	418 (56.3)	324 (43.7)	1
2	446	873	104 (23.3)	342 (76.7)	2.04 (1.77–2.36)	308	805	102 (33.1)	206 (66.9)	2.00 (1.67–2.39)
3+4	225	183	20 (8.9)	205 (91.1)	3.79 (3.18–4.50)	94	155	19 (20.2)	75 (79.8)	3.25 (2.52–4.19)
Not known	42	79	13 (31.0)	29 (69.0)	–	22	77	13 (59.1)	9 (40.9)	–

Lymphoma stage^b^	I	338	1663	232 (68.6)	106 (31.4)	0.35 (0.28–0.43)	306	1555	225 (73.5)	81 (26.5)	0.40 (0.31–0.51)
II	375	1656	225 (60.0)	150 (40.0)	0.46 (0.38–0.55)	357	1643	224 (62.7)	133 (37.3)	0.56 (0.46–0.68)
III	281	1065	142 (50.5)	139 (49.5)	0.68 (0.57–0.82)	262	1060	142 (54.2)	120 (45.8)	0.80 (0.65–0.99)
IV	893	2433	324 (36.3)	569 (63.7)	1	695	2360	319 (45.9)	376 (54.1)	1
Not fully staged	250	403	53 (21.2)	197 (78.8)	1.08 (0.91–1.28)	89	297	47 (52.8)	42 (47.2)	0.74 (0.53–1.02)

B-symptoms	No	1182	4284	579 (49.0)	603 (51.0)	1	939	4056	566 (60.3)	373 (39.7)	1
Yes	955	2932	397 (41.6)	558 (58.4)	1.15 (1.03–1.29)	770	2853	391 (50.8)	379 (49.2)	1.20 (1.04–1.39)

Deprivation (quintile)	1 (affluent)	466	1621	226 (48.5)	240 (51.5)	1.09 (0.92– 1.30)	383	1580	224 (58.5)	159 (41.5)	1.05 (0.85–1.31)
2	494	1716	233 (47.2)	261 (52.8)	1	402	1631	227 (56.5)	175 (43.5)	1
3	414	1395	178 (43.0)	236 (57.0)	1.18 (0.99–1.41)	325	1337	174 (53.5)	151 (46.5)	1.18 (0.94–1.46)
4	365	1180	162 (44.4)	203 (55.6)	1.17 (0.97–1.41)	283	1133	160 (56.5)	123 (43.5)	1.09 (0.87–1.38)
5 (deprived)	391	1290	172 (44.0)	219 (56.0)	1.15 (0.96–1.37)	312	1221	168 (53.8)	144 (46.2)	1.20 (0.96–1.50)
Not known	7	16	5 (71.4)	2 (28.6)	–	4	14	4 (100.0)	–	–

a Adjusted for all other factors in the table.

b See definitions in Box 1.

**Table 3:** Five year overall and relative survival estimates (95% Confidence Intervals) for all patients and those treated with first-line chemotherapy with curative intent: HMRN patients (≥18 years) diagnosed with DLBCL 2004–12 and followed until February 2015

		All patients		First line chemotherapy with curative intent	
		Overall survival	Relative survival	Overall survival	Relative survival
					
	Total	46.2 (44.0–48.4)	54.6 (52.1–57.0)	58.5 (56.1–60.9)	67.0 (64.3–69.6)

Age at diagnosis (years)	18–54	73.7 (68.8–77.9)	74.8 (69.9–79.0)	76.7 (72.0–80.8)	77.9 (73.1–82.0)
55–64	58.7 (53.3–63.6)	61.5 (55.9–66.7)	63.5 (57.9–68.5)	66.5 (60.6–71.7)
65–74	48.5 (44.4–52.5)	54.5 (49.9–58.9)	57.2 (52.7–61.3)	64.1 (59.1–68.6)
75–84	30.3 (26.4–34.3)	41.2 (35.9–46.5)	43.9 (38.7–49.0)	59.3 (52.1–65.9)
≥ 85	8.1 (4.9–12.3)	16.5 (9.9–24.6)	26.4 (15.6–38.6)	50.5 (27.1–69.9)

Sex	Males	46.5 (43.4–49.5)	54.9 (51.4–58.3)	57.2 (53.8–60.4)	66.2 (62.4–69.7)
	Females	45.8 (42.6–49.0)	54.2 (50.7–57.7)	60.0 (56.4–63.5)	67.9 (64.0–71.6)

Patient performance status^a^	0	75.0 (70.9–78.6)	86.6 (82.0–90.1)	76.5 (72.5–80.1)	87.1 (82.5–90.6)
1	53.2 (49.7–56.6)	62.8 (58.9–66.5)	60.7 (57.0–64.2)	69.6 (65.4–73.3)
2	20.9 (17.2–24.8)	25.5 (21.1–30.1)	33.6 (28.2–39.1)	39.1 (32.8–45.3)
3+4	3.2 (1.8–5.0)	3.9 (2.3–6.1)	20.4 (12.5–29.6)	22.9 (14.0–33.2)

Lymphoma stage^a^	I	71.5 (66.0–76.2)	84.5 (78.1–89.1)	75.5 (70.0–80.2)	86.4 (80.0–90.8)
II	64.3 (59.0–69.1)	73.9 (67.9–78.9)	67.4 (62.1–72.2)	76.5 (70.5–81.5)
III	53.2 (47.0–59.1)	61.6 (54.4–68.1)	57.6 (51.1–63.6)	66.3 (58.8–72.7)
IV	35.0 (31.8–38.2)	40.4 (36.9–44.0)	47.3 (43.5–51.1)	53.8 (49.5–57.9)
Not fully staged	14.7 (10.8–19.3)	21.6 (15.9–27.9)	53.8 (41.9–64.2)	66.9 (51.3–78.5)

Deprivation (quintile)	1 (affluent)	48.2 (43.4–52.9)	56.2 (50.8–61.3)	60.1 (54.7–65.0)	68.0 (62.2–73.2)
2	48.0 (43.4–52.5)	56.6 (51.3–61.5)	58.8 (53.6–63.6)	67.6 (61.9–72.7)
3	45.1 (40.1–49.9)	53.0 (47.4–58.4)	58.3 (52.6–63.5)	66.0 (59.7–71.6)
4	44.9 (39.6–50.0)	53.1 (46.9–58.9)	59.7 (53.5–65.3)	68.2 (61.3–74.2)
5 (deprived)	43.8 (38.6–48.9)	53.1 (47.0–58.9)	55.5 (49.5–61.1)	64.5 (57.5–70.6)

a See definitions in Box 1.
